# Thalassemia and Hemoglobinopathy Screening in Women Attending Antenatal Clinic at a Tertiary Care Center in Uttarakhand, India: A Re-look at the Laboratory Parameters Mandating High-Performance Liquid Chromatography Workup

**DOI:** 10.7759/cureus.40667

**Published:** 2023-06-19

**Authors:** Neha Singh, Nilotpal Chowdhury, Anupama Bahadur, Sana Ahuja, Kunnumbrath Arathi, Reshma Jeladharan, Anissa A Mirza, Arvind K Gupta, Harish Chandra, Shalinee Rao

**Affiliations:** 1 Pathology and Laboratory Medicine, All India Institute of Medical Sciences, Rishikesh, IND; 2 Obstetrics and Gynecology, All India Institute of Medical Sciences, Rishikesh, IND; 3 Oncopathology, Vardhman Mahavir Medical College & Safdarjung Hospital, New Delhi, IND; 4 Pathology, Employees' State Insurance Corporation (ESIC) Medical College and Post Graduate Institute of Medical Sciences and Research (PGIMSR), Chennai, IND; 5 Biochemistry, All India Institute of Medical Sciences, Rishikesh, IND

**Keywords:** thalassemia, hplc, hemoglobinopathies, hba2, β-thalassemia trait, antenatal screening

## Abstract

Introduction: Thalassemia and hemoglobinopathies are the most common inherited hematological disorders. Of these, β thalassemia is the commonest disorder reported in India, followed by certain hemoglobinopathies encountered in different regions of the country. The data pertaining to the incidence of these disorders in the Uttarakhand region of India are sparse.

Aim and objectives: To ascertain the prevalence and spectrum of thalassemia/hemoglobinopathies amongst antenatal women in Uttarakhand. The study also aimed to analyze the ability of red cell indices in differentiating beta thalassemia trait (BTT) from mild iron deficiency anemia (IDA).

Material and methods: A total of 460 pregnant women in the first trimester of pregnancy were screened by cation exchange high-performance liquid chromatography. Retention time and proportions of normal/abnormal hemoglobin peaks were documented in all cases. Hemoglobin A2 (HbA2) values of ≥4% were taken as a cut-off for diagnosing BTT. Blood samples were also collected for complete blood counts, reticulocyte counts, and serum ferritin. The ability of the various discriminatory indices to differentiate between IDA and BTT was also assessed.

Results: The prevalence of BTT and hemoglobin D-Punjab trait amongst pregnant women was found to be 2.6% and 0.2%, respectively. RBC count, mean corpuscular volume (MCV), and mean corpuscular hemoglobin (MCH) were found to be moderately strong predictors of BTT, with an area under the curve of 0.860, 0.857, and 0.842, respectively, which were comparable to the discriminatory indices found to be most useful in this study.

Conclusion: In view of the 2.6% prevalence of BTT in antenatal women in this region of Uttarakhand, a routine screening will be helpful in detecting carriers early in the antenatal period. Careful interpretation of red cell indices is crucial to the distinction between BTT and IDA. Discriminatory indices are reasonably accurate in differentiating BTT from mild iron deficiency, but for practical purposes, MCV and MCH provide equivalent information to identify cases that require further workup.

## Introduction

Thalassemia and hemoglobinopathies are one of the commonest inherited hematological disorders. According to the World Health Organization, almost 7% of the entire population of the world is a carrier for hemoglobin (Hb) disorders [1,2]. These disorders pose a major burden to the public healthcare system in many parts of the world, including India [1,3-6]. The most effective approach to overcome this problem is the implementation of a robust screening program at the population level. Persons diagnosed with these disorders should be provided with timely genetic counseling and offered necessary intervention [4].

These disorders occur worldwide, but there is a considerable variation in the prevalence between different regions of the world, and even between different areas within a country. The prevalence of these disorders is gradually showing an increasing trend in all parts of the world due to migratory populations [1,2]. β thalassemia is the most common thalassemia syndrome reported in India, along with some hemoglobinopathies being encountered primarily in certain regions/states of the country, like hemoglobin S (HbS) in Orissa, hemoglobin D (HbD) in Punjab, and hemoglobin E (HbE) in the north-eastern states [3,5]. The data pertaining to the prevalence and spectrum of Hb disorders in the Uttarakhand region of India are sparse, and a pertinent literature search revealed only a few published studies on the thalassemia syndromes and hemoglobinopathies that prevail in this region [7-10]. Since antenatal women are the group likely to benefit the most from screening for these inherited Hb disorders, therefore this study was conducted on pregnant women.

Aim and objectives

To ascertain the prevalence and spectrum of thalassemia/hemoglobinopathies amongst antenatal women in the Uttarakhand region of India by screening in the first trimester of pregnancy. The study also aimed to determine the role of various discriminatory indices in differentiating between beta thalassemia trait (BTT) and iron deficiency anemia (IDA).

## Materials and methods

This was a prospective cross-sectional study conducted at a tertiary care institute in Uttarakhand, India, which included 460 pregnant women attending the obstetrics outpatient department clinics. Women in the first trimester of pregnancy (till 12 completed weeks of gestation) were included in the study. Women who were taking iron supplements or had a recent history of blood transfusion were excluded from the study. The coronavirus disease 2019 (COVID-19) pandemic occurred during the course of the study; therefore, consecutive sampling could not be followed throughout and hence random sampling was done. The participants of the study were informed about the importance of screening for thalassemia/hemoglobinopathies, and informed written consent was taken from each patient. The study was started after obtaining approval from the Institutional Ethics Committee, AIIMS Rishikesh (approval no.: 74/IEC/IM/2017).

Four milliliters (ml) of blood was collected from each patient, 2 ml in ethylenediaminetetraacetic acid (EDTA) vacutainer for complete blood counts, reticulocyte count, and high-performance liquid chromatography (HPLC), and 2 ml in a plain vacutainer for serum ferritin estimation. The EDTA anticoagulated blood samples were run on Sysmex XN1000 automated hematology analyzer (Sysmex Corporation, Kobe, Japan) in the retic mode. The Hb, red cell count, red cell indices, reticulocyte count, and reticulocyte Hb content (Ret-Hb) were recorded for all cases. Serum ferritin levels were estimated in each case by the chemiluminescence method, using ADVIA Centaur XP semi-automated analyzer (Siemens Healthineers, Erlangen, Germany). Patients were categorized as IDA on the basis of a cut-off value of 15 ng/ml for serum ferritin as per the recommendations [11-13].

Cation exchange HPLC was performed on all the cases using the Bio-Rad D-10 automated HPLC system (Bio-Rad Laboratories, Hercules, CA). The retention time and proportions (%) of normal and/or abnormal Hb and peak characteristics for all Hb fractions were documented in all the cases. Hemoglobin A2 (HbA2) values of ≥4% were taken as the cut-off for diagnosing BTT [2,6,14]. In women who tested positive for thalassemia syndromes/hemoglobinopathies, the husbands were also screened, and genetic counseling was offered to the couple.

Since we wanted to identify discriminators that will help in differentiating between BTT and IDA with normal/near-normal Hb values, we took a cut-off Hb at ≥10 gm/dl and divided the patients into three groups: group 1: normal (no iron deficiency, no thalassemia) with Hb values > 10 gm/dL and ferritin > 15 ng/ml; group 2: mild IDA, with ferritin < 15 ng/ml; group 3: BTT (with or without iron deficiency) with HbA2 ≥ 4%. Using the RBC counts and red cell indices, various discriminant indices like England and Fraser, Green and King, Mentzer, red cell distribution width (RDW) index, Srivastava index, Sirdah, Shine & Lal, and Ricerca indices were calculated for each patient, using the described formulae [15-17].

Statistical analysis

Continuous variables were expressed as mean ± standard deviation (SD). Categorical variables were expressed as frequencies and percentages. Statistical analysis was carried out using the ‘R’ statistical software, version 4.0.3 (R Foundation for Statistical Computing, Vienna, Austria). The prevalence was calculated at a confidence level of ±2% and reported with a 95% confidence interval. The ability of the various discriminant indices to discriminate between IDA and BTT was calculated by confidence interval by the area under the curve (AUC) (method of deLong, computed by package pROC or R statistical software version 4.0.3). The receiver operating characteristic (ROC) curves of each of the discriminatory indices and RBC indices were also examined individually. A region of minimum acceptable diagnostic accuracy in the ROC curve was defined as the region where both sensitivity and specificity were greater than 0.7. The performance of the different indices in this region of acceptable accuracy was especially examined to identify which index has the highest sensitivity and specificity.

## Results

The age of the women ranged from 19 to 40 years, with a mean age of 26.9 ± 3.65 years (mean ± SD). The gestational age ranged from four to 12 weeks with a mean gestational age of 8.86 ± 2.67 weeks. Out of the 460 women screened, 12 had BTT, with HbA2 levels ≥ 4% on HPLC. One woman showed the presence of an abnormal Hb with a retention time of 3.97 seconds, which corresponded to HbD Punjab amounting to 36.8%, HbA0 of 52.2%, HbA2 of 2.8%, and HbF of <0.8% and was accordingly reported as HbD Punjab trait. The prevalence of BTT was thus 2.6% (12/460 cases), and that of the HbD Punjab trait was 0.2% (1/460 cases) amongst pregnant women. No other variants of Hb (like HbS, HbE, HbD, or other rarer variants) were detected amongst the antenatal women included in the study. The partners of the 13 women were offered screening by Hb HPLC, and all were found to be normal.

**Figure 1:**
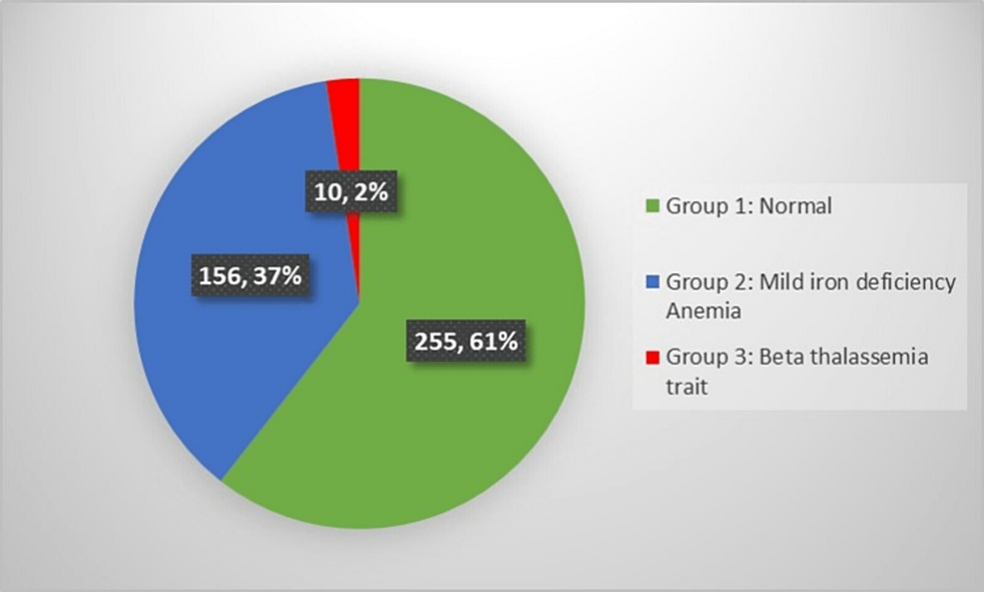
Distribution of 421 cases in three groups using a cut-off value of 10 gm/dl for hemoglobin levels.

Using a cut-off value of Hb of 10 gm/dl, 421 women were selected for further analysis. They were divided into three groups, as described above. Of these, 156 (37%) of the women had mild IDA. The distribution of cases in the three groups is shown in Figure 1. The remaining 39 women had a Hb value below 10 gm/dl and they were not included in the above groups, since we wanted to identify discriminators that will help in differentiating between BTT and iron deficiency with normal/near-normal Hb values. Therefore, these women were excluded from further analysis. The patients with mild anemia, who had BTT (group 3) showed microcytosis with mean corpuscular volume (MCV) of 72.07 ± 10.07 fl. The hematological parameters and red cell indices for three groups of participants are elaborated in Table 1. As expected, patients with BTT showed significantly higher levels of HbA2 as compared to the normal population, while patients with mild IDA showed lower HbA2 values (on HPLC) than the normal (Table 1). The mean serum ferritin level in cases of BTT (without IDA) in our study population (group 3) was 34.1 ng/ml. Three out of 12 (25%) patients with BTT were found to be iron deficient. The median and range of serum ferritin levels in the three groups are elaborated in Table 2.

**Table 1:** Hemoglobin, red cell indices, Ret-Hb, and HbA2 values in the three groups of participants. Values are expressed as mean ± standard deviation. Hb: hemoglobin; MCV: mean corpuscular volume; MCH: mean corpuscular hemoglobin; MCHC: mean corpuscular hemoglobin concentration; Ret-Hb: reticulocyte hemoglobin content; RDW-CV: red cell distribution width – coefficient of variation; HbA2: hemoglobin A2.

Parameter	Group 1: Normal (n = 255)	Group 2: Mild iron deficiency anemia (n = 156)	Group 3: Beta thalassemia trait (n = 10)
Hb (gm/dl)	12.46 ± 1.03	11.67 ± 0.92	11.38 ± 0.56
RBC count (million/ mm^3^)	4.26 ± 0.42	4.22 ± 0.42	5.09 ± 0.65
MCV (fL)	89.19 ± 5.43	86.00 ± 7.17	72.07 ± 10.08
MCH (pg)	29.36 ± 2.09	27.84 ± 2.80	22.79 ± 3.87
MCHC (g/dl)	32.91 ± 0.98	32.29 ± 1.20	31.52 ± 1.08
RDW-CV	14.07 ± 1.36	14.96 ± 1.82	15.94 ± 1.22
Ret-Hb	31.24 ± 2.52	29.33 ± 3.16	24.51 ± 3.90
HbA2	3.18 ± 0.32	3.06 ± 0.38	5.42 ± 1.00

**Table 2:** Median and range of serum ferritin levels in the three groups. IDA: iron deficiency anemia; BTT: beta thalassemia trait.

Group	Description	N	Mean ferritin value (ng/ml) (range)
1	Group 1: Normal	255	32.6 (15.3-247.2)
2	Group 2: Mild IDA	156	9.0 (0.3-14.8)
3	Group 3: Only BTT	7	34.1 (17.5-197.4)
	Group 3: BTT with mild IDA	3	7.8 (7.8-12.1)

The discriminant indices were calculated (as per the formulae described) for all the 421 cases included in the subsequent analysis. The ability of the various discriminant indices to discriminate between mild IDA and BTT was calculated by confidence interval by the AUC. England and Fraser's index was found to be the most useful index in differentiating between mild IDA and BTT, with an AUC of 0.868, followed by the Sirdah Index with an AUC of 0.867 and Mentzer index with an AUC of 0.865 (Table 3).

**Table 3:** Comparison of various discriminatory indexes in differentiating iron deficiency anemia from beta thalassemia trait. Hb: hemoglobin; IDA: iron deficiency anemia; BTT: beta thalassemia trait; MCV: mean corpuscular volume; RBC: red blood cell count; RDW: red cell distribution width; MCH: mean corpuscular hemoglobin.

Name of index	Formula	Established cut-off values		Area under the curve	Confidence interval
		BTT	IDA		
England and Fraser	MCV - (5 × Hb) - RBC - 3.4	<0	>0	0.868	0.744-0.993
Sirdah index	MCV - RBC - (3 × Hb)	<27	>27	0.867	0.730-1
Mentzer index	MCV/RBC	<13	>13	0.865	0.721-1
Shine & Lal index	MCV × MCV × MCH/100	<1530	>1530	0.852	0.690-1
Srivastava index	MCH/RBC	<3.8	>3.8	0.855	0.701-1
Green and King	MCV × MCV × RDW/Hb × 100	<65	>65	0.839	0.704-0.975
RDW index	MCV × RDW/RBC	<220	>220	0.843	0.699-0.987
Ricerca index	RDW/RBC	<4.4	>4.4	0.748	0.598-0.899

We also analyzed the ability of red cell indices in predicting BTT using ROC, and calculated the AUC and confidence interval for each, as shown in Table 4. We found that RBC count and MCV were moderately strong predictors of BTT, with an AUC of 0.860 and 0.857, respectively, which are comparable to the discriminatory indices like England and Fraser, Mentzer index, and Sirdah index, found to be most discriminatory in the present study. We found preliminary cut-offs of 73 fL for MCV, 4.82 million/mm3 for RBC count, and 24.65 pg for mean corpuscular hemoglobin (MCH), which were found to be helpful in differentiating BTT from IDA. The sensitivity and specificity of MCV, MCH, and RBC count at the proposed cut-offs outperform the best-performing indices at the pre-defined cut-offs of the discriminatory indices (Table 5). While the Shine & Lal index has slightly increased sensitivity compared to MCV, it has decreased specificity resulting in almost equal to Youden’s J. At pre-defined cut-offs for the discriminatory indices, the diagnostic performance of the formula-based indices other than Shine & Lal showed marked degradation compared to the optimal cut-offs from ROC curve analysis. This may possibly be due to the different populations (pregnant females) being studied presently, as compared to the populations on which these indices were validated. This suggests that we need new cut-offs for these indices in pregnant females. In most cases, the sensitivity is decreased, except for the Ricerca index, which shows high sensitivity, but is diagnostically not useful due to specificity being less than 0.1. But then again, due to the low number of BTT cases, and an even smaller number of BTT cases having a concomitant iron deficiency, these cut-offs have limited reliability at present.

**Table 4:** Comparison of red cell indices in differentiating iron deficiency anemia from beta thalassemia trait. AUC: area under the curve; CI: confidence interval; RBC: red blood cell; MCV: mean corpuscular volume; MCH: mean corpuscular hemoglobin; MCHC: mean corpuscular hemoglobin concentration; RDW-CV: red cell distribution width – coefficient of variation; Ret-Hb: reticulocyte hemoglobin content.

Red cell indices	AUC (CI)
RBC count	0.860 (0.725-0.995)
MCV	0.857 (0.700-1)
MCH	0.842 (0.671-1)
MCHC	0.711 (0.539-0.883)
RDW-CV	0.717 (0.571-0.864)
Ret-Hb	0.823 (0.647-0.999)

**Table 5:** Comparison of sensitivity and specificity of formula-based discriminatory indices at pre-defined cut-offs and red cell indices at the proposed cutoffs. RDWI: red cell distribution width index; MCV: mean corpuscular volume; MCH: mean corpuscular hemoglobin; RBC: red blood cells.

Discriminatory index/red cell parameter	Cut-off	Sensitivity	Specificity	Youden’s J
England and Fraser	0	0.3	1	0.3
Sirdah	27	0.4	0.99	0.39
Mentzer	13	0.5	0.99	0.49
Shine & Lal	1530	0.8	0.85	0.65
Srivastava	3.8	0.4	0.98	0.38
Green & King	65	0.5	0.99	0.49
RDWI	220	0.6	0.97	0.57
Ricerca	4.4	1	0.08	0.08
MCV	73	0.7	0.96	0.66
MCH	24.65	0.8	0.88	0.68
RBC count	4.815	0.7	0.9	0.6

**Figure 2:**
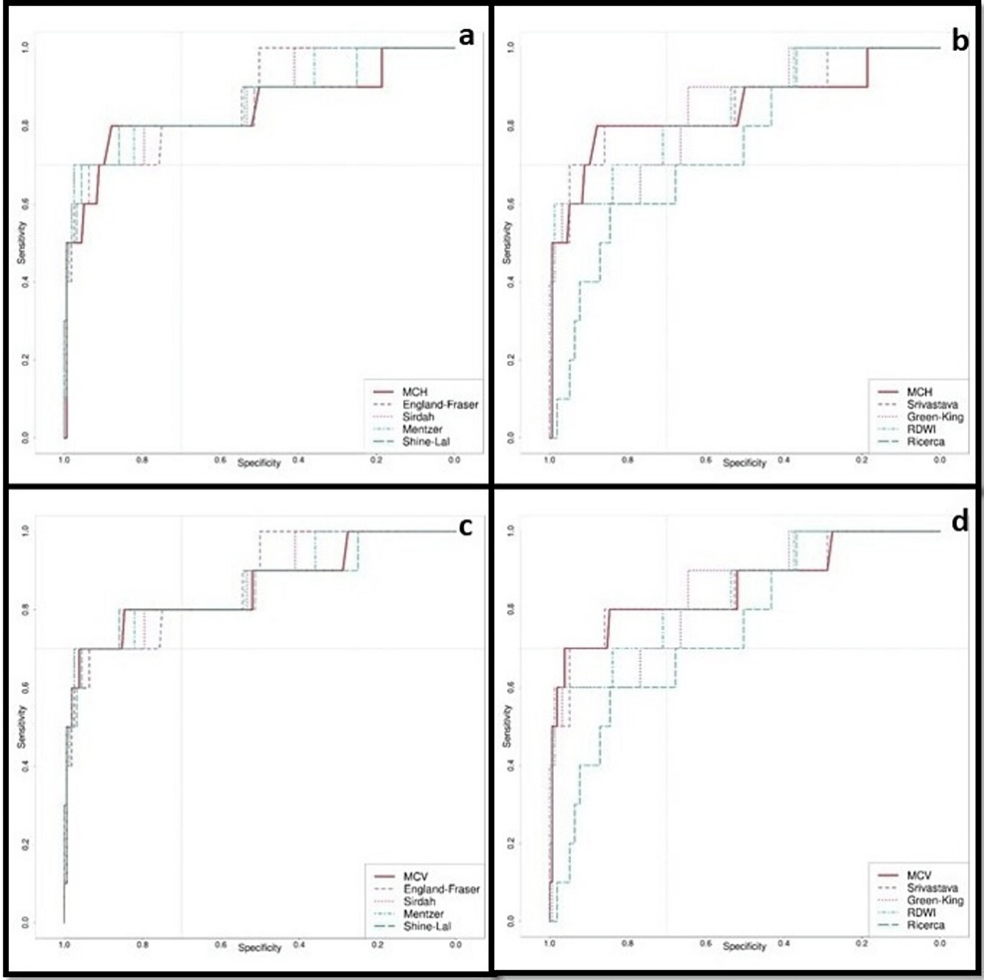
Comparison of the ROC curve of MCV with the different indices (a-b) and MCH with the different indices (c-d). The area of minimum acceptable accuracy was defined as the area having both sensitivity and specificity greater than 0.7 (marked by dotted gray lines): the top-left square. ROC: receiver operating characteristic; MCV: mean corpuscular volume; MCH: mean corpuscular hemoglobin; RDWI: red cell distribution width index.

The ROC curves of MCV, MCH, and the formula-based discriminatory indices were also examined (Figure 2). The relevant area of high diagnostic accuracy of the ROC plot (areas where both the sensitivity and specificity were high) were examined for differences. For the range of specificities from 0.7 and above, the maximum sensitivity reached for the most accurate discriminatory indices was 0.8, achieved by the England and Fraser, Sirdah, Mentzer, Shine & Lal, Srivastava, and RDWI indices as well as MCV and MCH. A higher sensitivity was achieved only at cut-offs, which resulted in lower, unsatisfactory specificities. Among all the indices, MCH was the best-performing index in the region of the ROC curve corresponding to acceptable sensitivity and specificity (the region where both sensitivity and specificity were greater than 0.7), achieving the aforementioned sensitivity combined with the highest specificity in this region. In the region of acceptable accuracy, MCH was followed by the Shine & Lal and Srivastava indices, which performed equally, followed by MCV and then other indices. The differences between the Shine & Lal index, Srivastava index, and MCV were minor and essentially equivalent (a maximum difference of 0.013 for specificity for the same sensitivity in the area corresponding to satisfactory diagnostic performance of the ROC curves). Other indices achieved a higher AUC for the ROC because they were associated with higher sensitivity at low specificity values.

## Discussion

Hereditary disorders of Hb are categorized into two main groups: hemoglobinopathies, which are characterized by the production of structurally abnormal globin chains, and thalassemia syndromes, which feature a decreased production of normal globin chains. The inherited Hb disorders pose a considerable healthcare burden in India, with β thalassemia, HbS, HbD, and HbE being the most commonly reported variants [18,19]. The reported prevalence of β thalassemia in India ranges from 3% to 4%, with a much higher prevalence amongst certain ethnic populations like Punjabis, Sindhis, Mahars, Lohanas, Saraswats, Bhanushalis, Parsis, Gujaratis, and Bengalis [2,20,21]. According to a recent study, the overall prevalence of BTT/β thalassemia minor in India is 2.78% with a range of 1.48-3.64% amongst different regions of the country [22]. HbS is prevalent in Orissa, Madhya Pradesh, Andhra Pradesh, the Chetti tribes of Kerala, the Nilgiri region of Tamil Nadu, and Maharashtra. HbE is found in eastern states, mainly in Assam, Bihar, and West Bengal, where the carrier frequency ranges from around 3% to nearly 50% [2,20].

There is a lack of substantial data pertaining to the prevalence of Hb disorders in the Uttarakhand region of India. Only a few published studies from this region are available in the literature, which are unable to comprehensively reflect the magnitude of the problem in this hilly state [7-9]. Nayar et al. [7] screened 8144 individuals from the Garhwal region and found BTT to be the commonest of these disorders here, with an incidence of 2.82%, followed by HbD Punjab (0.55%) and HbE trait (0.42%). Mishra et al. [8] screened 920 randomly selected individuals from the Garhwal region of Uttarakhand for BTT by naked eye single tube red cell osmotic fragility test (NESTROFT) first, followed by HbA2 estimation. They reported the incidence of BTT to be 1.5%, using a cut-off of 3.5% for HbA2. Malik et al. [9] reported a 4.2% prevalence of hemoglobinopathies in the state [9]. In the present study, we found a 2.6% incidence of BTT and 0.2% of HbD Punjab trait amongst antenatal women in this region. We did not encounter any other hemoglobinopathy or thalassemia syndrome amongst the pregnant women screened in this study.

Iron deficiency anemia is very common in antenatal women commonly. BTT needs to be differentiated from IDA since both of them have microcytic hypochromic red cells. Hence, BTT may often be mistaken for IDA and the woman may be prescribed unnecessary iron supplements [4]. HbA2 estimation by HPLC readily helps in resolving this dilemma, but in resource-limited settings, this facility may not be readily available. In such situations, one has to majorly depend on the interpretation of red cell parameters and various predictive/discriminatory indices described, to pick up probable cases of BTT, which require further workup by HPLC and/or further ancillary tests [15,16].

Various discriminatory indexes are calculated based on certain formulas and are used as predictive indices to differentiate BTT from IDA. However, it has been seen that none of these indices is 100% sensitive or specific in differentiating BTT from IDA [15]. In the present study, we evaluated a compendium of various discriminatory indices and red cell parameters to identify which of these were practically the most helpful ones. The role of RBC counts in differentiating between BTT and IDA is well established, and our results also confirmed the same. Apart from that, an interesting finding that emerged in our study was that MCV was found to be a moderately strong predictor of BTT, with an AUC of 0.8567 and CI of 0.7-1, closely followed by MCH with an AUC of 0.842, which is similar to the other most specific discriminatory indices like England and Fraser, Mentzer index, and Sirdah index (Table 3). MCV and MCH are easily available red cell parameters, provided by all automated hematology cell counters. Due to the equivalent performance of MCV and MCH compared to the discriminatory indices for satisfactory diagnostic accuracy, there seems to be little additional predictive information that these indices can provide over and beyond the easily available MCV and MCH.

The results of our study highlight that none of the discriminatory indices is likely to be more useful than the easily available red cell parameters like MCV and MCH. We found these simple parameters to be practically equivalent to some of the most reliable discriminant indices, which require calculations and memorizing complex formulae. Since both these parameters and discriminant indices are only reliable "indicators" to identify cases that require workup for BTT, it raises a pertinent question of whether one needs to go through the extra effort to calculate the discriminatory indices. We could, however, not establish reliable cut-offs for MCV and MCH, for levels above/below which they have the most reliable discriminatory power, due to the small number of BTT cases in this study, and also because some of the cases had BTT combined with IDA. A study with a larger sample size would be required to determine the optimal cut-offs.

Screening for BTT is crucial since thalassemia/hemoglobinopathies pose a serious socioeconomic burden on the community due to transfusion dependence and high costs incurred in treatment and iron chelation [2]. The problem is especially worrisome in India since the literacy rate in our country is low, consanguineous marriages are still rampant in some regions of India, and screening for these disorders is not being performed uniformly. It has to be reiterated that a complex disease like thalassemia can easily be prevented if screening of antenatal women and couples at risk is universally implemented. Therefore, the implementation of a community-based control program will be the most cost-effective approach to reducing the burden of this disease [19,22]. The BTT/carriers or affected individuals should be offered genetic counseling, prenatal diagnosis, and the option of selective termination of the affected fetus, thereby preventing the birth of a thalassemic child [4].

Antenatal screening for thalassemia syndromes/hemoglobinopathies is gradually gaining pace in our country and is now being done in many centers. HPLC is a rapid, dependable, and reproducible technique for the identification of HbA2, HbF, and various abnormal Hb, and has consequently emerged as the method of choice for screening for Hb disorders [14]. Elevated HbA2 level is a well-established screening test for BTT. However, there is no clearly defined consensus on the cut-off value of HbA2 for identifying BTT [23]. Many centers use an HbA2 value of ≥3.5% for the diagnosis of BTT, as recommended by a few authors and textbooks [4,23], while others have recommended a cut-off of ≥4% and documented a higher sensitivity for detecting cases of BTT at this value [1,6,14,23,24]. In the present study, we used a cut-off of 4% for HbA2 for diagnosing BTT. It is recommended that serum ferritin estimation should be an integral part of screening of hemoglobinopathies since IDA can lower HbA2 values and the same was followed in our study [6].

Despite all the advantages of HPLC, routine testing through the HPLC system for all antenatal women can prove to be a financial burden for our country’s healthcare system. Therefore, in resource-limited settings, one has to judiciously identify cases that require further workup through HPLC and molecular techniques to detect these Hb disorders. Consequently, reliable criteria based on routine laboratory parameters are essential, which may be considered significant in mandating HPLC workup. This will prove to be a feasible, practical as well as cost-effective approach to screening and diagnosing thalassemia syndromes/hemoglobinopathies in antenatal women.

Limitations of the study

The number of cases in this study is low because the COVID-19 pandemic occurred during the course of the study and hence consecutive sampling could not be done. Due to the small number of cases with BTT, we could not establish reliable cut-offs for MCV for levels above/below which it has the most reliable discriminatory power. A study with a larger sample size would be required to determine the optimal cut-offs.

## Conclusions

The prevalence of BTT in antenatal women in the Uttarakhand region of India was found to be 2.6%, while that of the HbD Punjab trait was 0.2% on screening with Hb HPLC. In view of this, a routine screening will be helpful in detecting carriers early in the antenatal period in this region. In resource-limited settings, careful interpretation of the red cell indices is crucial to the distinction between BTT and IDA, and to identify cases that require further workup via HPLC. In the present study, MCV and MCH were found to be moderately strong predictors of BTT. Formula-based discriminatory indices are reasonably accurate in differentiating BTT from mild iron deficiency. However, the role of simple red cell indices like MCV and MCH is comparable to the most reliable discriminatory indices, and these parameters provide equivalent information to identify cases that require further workup.
